# Investigation of symptom management and functional state of women who underwent breast cancer surgery

**DOI:** 10.1590/1806-9282.20230954

**Published:** 2024-03-15

**Authors:** Murat Can Mollaoğlu, Esra Başer Akın, Mukadder Mollaoğlu, Kürşat Karadayı

**Affiliations:** 1 Sivas Numune Hospital, Oncological Surgery Clinic – Sivas, Turkey.; 2 Sivas Cumhuriyet University, Faculty of Health Science, Department of Nursing – Sivas, Turkey.; 3 Sivas Cumhuriyet University, Oncological Surgery Clinic – Sivas, Turkey.

**Keywords:** Breast cancer, Disease management, Functional status, Symptom cluster, Health status, Mastectomy

## Abstract

**OBJECTIVE:**

The aim of this study was to investigate the symptom management and the functional status of women who underwent surgery for breast cancer.

**METHODS:**

This cross-sectional descriptive study was conducted in a university hospital surgical oncology clinic. This study was conducted on 80 patients who had undergone breast cancer surgery in the last 5 years in a surgical oncology clinic of a university hospital. Study data were collected using the patient identification form, Symptom-Management Self-Efficacy Scale Related to Chemotherapy in Breast Cancer, and Functional Living Index-Cancer. The data were analyzed with the SPSS program.

**RESULTS:**

The mean total score of Symptom-Management Self-Efficacy Scale Related to Chemotherapy in Breast Cancer was found to be 157.28±36.86, and the mean total score of the Functional Living Index-Cancer was found to be 103.79±18.77. When the correlation between the Functional Living Index-Cancer and Symptom-Management Self-Efficacy Scale Related to Chemotherapy in Breast Cancer scales used in the study was examined, it was determined that there was a positive statistically significant correlation (p<0.05) between the subscale and scale total scores.

**CONCLUSION:**

As a result of the study, it was determined that the self-efficacy and functional status of the patients were poor. Their functional status was also determined to be improved as the symptom self-efficacy levels increased.

## INTRODUCTION

Although surgical interventions are effective in treating cancer, treatment can often result in side effects that can affect the patient's function and quality of life (QoL). Even in the years after treatment, a functional decline is observed in patients^1^. Regardless of the type of surgical intervention, the functional status may be affected in patients in the following years due to pain, limitation of movement in the arms, and edema formation^2^. Functional status is a complex, multidimensional assessment of individuals’ physical, psychological, and social well-being. Physical dimensions, work, and physical functionality include the ability to cope with psychological dimensions, self-acceptance, perceived health status, and adaptation to the disease^3^.

Symptoms that develop in patients can negatively affect their QoL and functional status^4^. For this reason, it is thought that routine follow-ups will increase the QoL and functional independence of patients, as they provide the opportunity for symptom recognition and early intervention^1,4,5^. In addition, cancer reduces the individual's ability to cope with the disease, depending on the symptoms it causes in individuals. Thus, the physical, emotional, and social well-being of the patients is affected; they may feel powerless; and their self-efficacy perceptions may be negatively affected.

Self-efficacy is considered an effective component of well-being and successful symptom management. Self-efficacy, which plays an important role in facilitating health behavior and therefore improving health outcomes, is extremely important for cancer patients to cope with their symptoms and adapt to the process in their functional lives^5,6^. When the literature is examined, it has been observed that there are few studies examining self-efficacy and functional status in symptom management in patients after breast surgery^1,5-8^. In fact, no study that considers the two variables together has been found. The aim of this study was to examine symptom management and functional status of women who underwent breast cancer surgery and to evaluate the relationship between symptom management and functional status.

## METHODS

This cross-sectional study was conducted in a university hospital surgical oncology clinic. Patients who had undergone surgery for breast cancer in the surgical oncology department in the last 5 years were included in the study. In the surgical oncology clinic, patient records were examined, and patients who met the criteria were reached. Inclusion criteria for the study were as follows: having received at least two cures of chemotherapy or two cures of radiotherapy, volunteering to participate in the study, being 18 years of age or older, and not having a health problem that prevents communication.

In this study, when α=0.05, β=0.10, and 1-β=0.90 were calculated, 80 people were included in the study. The power of the test was obtained as p=0.90104 (PASS 2011 Home-Power Analysis and Sample Size)^9^.

Research data were collected through the forms described below.

### Patient information form

The demographic information about the patients and the data about the disease/treatment process were collected with a questionnaire form consisting of 21 questions prepared by using the literature.

### Symptom Management Self-Efficacy Scale Related to Chemotherapy in Breast Cancer

In Liang et al.'s study, the SMSES-BC was developed to evaluate symptom management and self-efficacy in patients with breast cancer undergoing chemotherapy^10^. This scale consists of three subdimensions and 27 items: problem-solving skills (seven items), management of symptoms related to chemotherapy (15 items), and management of emotional interpersonal problems (five items). The score for the entire scale ranges from 0 to 270. The high score indicates that the individual's perceived self-efficacy in managing symptoms is high. Its validity and reliability in Turkish were studied by Semiz and Sağlam, and the Cronbach's α coefficient was found to be 0.90^11^.

### Functional Living Index-Cancer

Functional Living Index-Cancer was developed by Schipper et al., for use in cancer patients^12^. The scale consisting of 22 questions was prepared according to the 7-item Likert scale. The FLIC has five subtitles: Physical Functions, Psychological Functions, General Wellbeing (Cancer-related Challenges), Social Functions, and Gastrointestinal Symptoms. The lowest score that can be obtained from the scale is 22, and the highest score is 154. Low scores indicate poor functional status and QoL. The scale's validity and reliability in Turkey were studied by Bektas and Akdemir^13^.

### Statistical analysis

Descriptive statistics and correlational analyses were conducted. The SPSS version 18.0 (Statistical Package for the Social Sciences, Chicago, IL, USA) program was used to evaluate the data obtained as a result of the research, and the number, percentage, mean, standard deviation, t-test, Mann-Whitney U test, and Kruskal-Wallis test were used.

Permission to conduct the study was obtained from the SCU Research Ethics Committee (decision no.: 2020-03/18). The study was conducted in accordance with the Declaration of Helsinki.

## RESULTS

Participant characteristics and descriptive statistics are displayed in Table 1. Of the patients included in the study, 66.2% were between the ages of 45 and 65 years, 63.8% were married, 52.5% were primary school graduates, 36.2% had stage 2 cancer disease, and 67.5% had mastectomy (Table 1).

**Table 1:** Comparison of sociodemographic characteristics and Symptom-Management Self-Efficacy Scale Related to Chemotherapy in Breast Cancer total and subscales.

Characteristics		n (%)	Problem-solving skills	Managing symptoms	Managing emotional interpersonal problems	Total
			X±S.D	X±S.D	X±S.D	X±S.D
Age (years)	20–45	10 (12.5%)	48.30±10.83	92.60±21.66	27.80±6.25	168.70±31.93
	45–65	53 (66.2%)	41.69±13.29	95.20±21.55	24.32±7.40	161.23±38.29
	65 and above	17 (21.2%)	31.17±10.87	83.29±17.49	23.76±5.19	138.24±29.17
	Statistical test		KW=12.68; p=0.002	KW=5.03; p=0.081	KW=2.39; p=0.303	KW=6.24; p=0.044
Marital status	Married	51 (63.8%)	42.96±12.40	96.11±21.97	26.17±6.79	165.25±36.83
	Single	29 (36.2%)	35.58±14.13	85.72±17.90	21.93±6.31	143.24±33.05
	Statistical test		t=2.42; p=0.017	t=-2.16; p=0.033	t=2.75; p=.007	t=2.66; p=0.009
Education	Illiterate	22 (27.5%)	28.09±9.07	78.36±17.80	21.72±5.36	128.18±24.82
	Primary school	42 (52.5%)	44.30±11.25	98.97±19.09	25.85±7.64	169.14±34.94
	High school	9 (11.2%)	42.44±13.38	89.88±24.78	24.11±6.43	156.44±37.33
	University	7 (8.8%)	51.71±13.07	99.71±18.35	27.14±4.59	178.57±30.26
	Statistical test		KW=27.70; p=0.001	KW=15.54; p=0.001	KW=6.42; p=0.093	KW: 21.71; p=0.001
Cancer stage	Stage 1	27 (33.8%)	43.62±11.64	97.03±13.74	27.11±7.04	167.78±28.35
	Stage 2	29 (36.2%)	40.20±13.88	91.72±26.17	23.96±6.63	155.90±43.71
	Stage 3	24 (30%)	36.62±14.35	87.83±20.78	22.66±6.45	147.12±34.54
	Statistical test		KW=3.16; p=0.206	KW=3.13; p=0.206	KW=3.84; p=0.147	KW=5.11; p=0.078
Type of surgery	Breast-conserving surgery	26 (32.5%)	43.69±12.21	96.19±13.90	27.11±6.91	167.00±29.45
	Mastectomy	54 (67.5%)	38.64±13.81	90.50±23.67	23.44±6.62	152.59±39.34
	Statistical test		U=560.50; p=0.146	U=576.50; p=0.197	U=531.50; p=0.79	U=511.00; p=0.050

X: mean; S.D: standard deviation; KW: Kruskal-Wallis; t: t-test; U: Mann-Whitney U test.

Symptom-Management Self-Efficacy Scale Related to Chemotherapy in Breast Cancer total score was evaluated as 157.28±36.86, and the FLIC total score was evaluated as 103.79±18.77.

As a result of comparing the sociodemographic characteristics of the patients with the SMSES-BC, the problem-solving subdimension and scale total scores of the patients aged 20–45 years were found to be statistically significantly higher (p<0.05). All subdimensions and total scores of those whose marital status was married were found to be high. The problem-solving, symptom management, and scale total score of the patients whose educational status was university and above were found to be statistically significantly higher (p<0.05). The scale total score of the patients who underwent breast-conserving surgery was found to be significantly higher (p<0.05). The management of symptoms subdimension and scale total scores were found to be statistically significantly higher (Table 1).

Table 2 shows the relationship between some sociodemographic characteristics of the patients and their functional life scale and subdimension means. According to the findings, the physical function subscale and scale total scores of the patients with an age range of 20–45 years were found to be statistically significantly higher. Physical, social function, general well-being subscale, and scale total score of the patients with university and higher education levels were found to be significantly higher than other education levels (p<0.05). The scale total score of the patients with stage 1 was evaluated to be higher than the patients in other stages. Physical function subdimension and scale total scores of patients who underwent breast-conserving surgery were found to be statistically significantly higher than those who underwent mastectomy (Table 2).

**Table 2:** Comparison of sociodemographic characteristics and Functional Life Scale-Cancer total and subdimension mean scores.

Characteristics		Physical functions	Psychological functions	General well-being	Social functions	Gastrointestinal symptoms	Total
		X±S.D	X±S.D	X±S.D	X±S.D	X±S.D	X±S.D
Age (years)	20–45	45.60±6.44	32.30±3.12	15.30±3.74	11.70±1.94	10.50±2.54	115.40±13.19
	45–65	39.01±10.42	29.52±5.46	13.83±4.14	10.62±2.60	10.90±2.29	103.91±19.76
	65 and above	34.47±8.66	29.23±4.71	12.70±3.63	10.88±2.11	9.29±3.01	96.58±15.25
	Statistical test	KW=8.05; p=0.018	KW=2.24; p=0.326	KW=3.59; p=0.166	KW=1.70; p=0.427	KW=3.77; p=0.152	KW=7.36; p=0.025
Marital status	Married	40.09±9.34	30.62±4.66	13.92±3.92	11.05±2.07	10.49±2.62	106.20±16.12
	Single	36.72±11.07	28.37±5.61	13.51±4.21	10.37±2.95	10.55±2.44	99.55±22.37
	Statistical test	t=−1.45; p=0.151	t=1.92; p=0.058	t=0.431 p=.668	t=1.20; p=0.232	t=−0.103; p=.918	t=1.53; p=0.129
Education	Illiterate	32.31±8.49	27.63±4.86	12.31±3.25	10.13±1.90	10.27±2.83	92.68±15.39
	Primary school	40.90±9.90	30.52±5.26	14.26±4.20	10.90±2.70	10.45±2.58	107.05±19.22
	High school	39.66±7.03	30.22±4.81	12.66±4.09	10.11±1.96	10.88±2.26	103.56±14.24
	University	46.28±9.69	31.85±3.89	16.85±3.07	13.28±0.75	11.14±1.95	119.43±14.22
	Statistical test	KW=14.65; p=0.002	KW=5.81; p=0.121	KW=8.98; p=0.029	KW=15.63; p=0.001	KW=0.573; p=0.903	KW: 15.86; p=0.001
Cancer stage	Stage 1	42.00±8.52	31.44±4.22	14.74±3.51	11.40±1.86	10.40±2.34	110.00±13.77±13.77
	Stage 2	38.79±10.43	29.27±5.07	14.34±3.68	10.86±2.50	10.44±3.00	103.72±20.11
	Stage 3	35.45±10.47	28.62±5.76	12.00±4.47	10.08±2.79	10.70±2.35	96.87±20.19
	Statistical test	KW=5.67; p=0.059	KW=4.03; p=0.133	KW=5.60; p=0.061	KW=23.25; p=0.197	KW=0.204; p=0.903	KW=6.89; p=0.032
Type of surgery	Breast-conserving surgery	42.88±8.00	31.26±4.40	14.84±3.39	11.46±1.74	10.61±2.24	118.08±13.44
	Mastectomy	36.94±10.44	29.11±5.31	13.25±4.21	10.50±2.66	10.46±2.69	100.28±20.03
	Statistical test	U=460.50; p=0.013	U=539.00; p=0.093	U=555.00; p=0.130	U=562.50; p=0.147	U=695.0; p=0.942	U=441.50; p=0.007

KW: Kruskal-Wallis, t: t-test, U: Mann-Whitney U test.

When the correlation between the FLIC and SMSES-BC scales used in the study was examined, it was determined that there was a positive statistically significant correlation (p<0.05) between the subscale and scale total scores. As the level of self-efficacy in managing symptoms increases, functional life capacity and QoL also increase (Table 3).

**Table 3:** The relationship between Symptom-Management Self-Efficacy Scale Related to Chemotherapy in Breast Cancer and Functional Living Index-Cancer.

FLIC/SMSES-BC		Physical functions	Psychological functions	General well-being	Social functions	Gastrointestinal symptoms	Total
Problem-solving skills	r	0.685**	0.447**	0.642**	0.323**	0.205	0.685**
	p	0.001	0.001	0.001	0.003	0.068	0.001
Managing symptoms	r	0.612**	0.384**	0.536**	0.371**	0.222*	0.626**
	p	0.001	0.001	0.001	0.001	0.048	0.001
Managing emotional interpersonal problems	r	0.613**	0.446**	0.560**	0.143	0.143	0.608**
	p	0.001	0.001	0.001	0.206	0.204	0.000
Total	r	0.708**	0.466**	0.645**	0.357**	0.228*	0.722**
	p	0.001	0.001	0.001	0.001	0.042	0.000

FLIC: Functional Living Index-Cancer; SMSES-BC: Symptom-Management Self-Efficacy Scale–Breast Cancer related to chemotherapy. ** and * indicate statistically significant values:

* p<0.01

** p<0.05.

## DISCUSSION

In this study, which included women who had undergone breast cancer surgery, it was determined that symptom management and functional status were adversely affected. Similarly, studies on different populations of patients who have been operated on with breast cancer have determined that diagnosis and treatment negatively affect patients’ self-efficacy and well-being^3,7^. Evaluation of patients’ self-efficacy and functional status is one of the determining parameters of QoL and is an important parameter that clinical oncology attaches importance from past to present^1,6^. It has been reported that education and counseling on health promotion strategies can improve patients’ self-efficacy and general well-being^4,6,8^. Increased self-efficacy not only helps patients effectively manage psychosocial variables associated with QoL but can also contribute to better compliance, positive treatment outcomes, and more informed treatment options.

In this study, the relationship of some sociodemographic and clinical characteristics of women with breast cancer with symptom management self-efficacy and functional status was also examined. Accordingly, symptom management self-efficacy was determined by age, marital status, educational status, and type of surgical intervention, whereas functional status was related to age, educational status, stage of the disease, and type of surgical intervention.

Self-efficacy and general well-being of women aged 20–45 years, the youngest group of the study, was found to be higher than other age groups in this study. Studies have reported that functional status regresses in advancing ages, depending on other increases in self-care activities, and therefore self-efficacy perception is negatively affected^8,14^. This finding is remarkable for monitoring and rehabilitating the functional status of elderly women who have undergone breast surgery. Thus, it can be ensured that older women get help earlier.

It was found that individuals whose marital status was married had higher symptom management self-efficacy levels in the study. Marriage, spouses providing support, and helping each other seem to be effective in managing symptoms, problem-solving, and emotional problems. In addition, this result can be explained by the strong social support of individuals^14^. When the literature was reviewed, it was determined that similar results were obtained in the studies^5,7^.

Early diagnosis of cancer is effective in preventing metastasis, increasing the quality of life, and prolonging life expectancy. Surgical interventions determined according to the stage of cancer also have different effects on survival. A recent retrospective cohort study clearly demonstrated the negative impact of advanced cancer on survival outcomes^15^. In another randomized controlled study, it was determined that breast magnetic resonance imaging, due to its high sensitivity in detecting invasive neoplasms, detects cancer at an early stage and has a role in prolonging life^16^. As determined in this study and in a similar study^5^, the functional status of survivors of early-stage breast cancer is better than those of advanced-stage breast cancer. For this reason, to increase the life expectancy and functional status of breast cancer patients, awareness of current diagnosis and treatment methods should be high, and these factors should be evaluated together with well-being.

In this study, FLIC and SMSES-BC scores were higher in patients who underwent breast-conserving surgery as a surgical intervention. In a study evaluating the QoL in women who underwent breast-conserving surgery and mastectomy, it was found that women who underwent mastectomy were more functionally affected^17^. One of the recent systematic reviews and meta-analysis, studies has brought to the attention of breast reconstruction surgeons that autologous fat grafting as a surgical intervention is a safe procedure in breast cancer and its role in improving both the survival and functional status of patients^18^. In other studies, it was found that the self-efficacy levels of women who had breast-conserving surgery were better^4,19^. This result is thought to be related to the effects of body image and functional capacity in women with mastectomy.

Studies have shown that individuals with high self-efficacy are more effective in managing the disease process and its treatments^4-8,19^. Similarly, in this study, as self-efficacy in symptom management increases, well-being in functional life increases. Therefore, it should be noted that high self-efficacy in patient care plays an important role in symptom management and improves functional well-being.

## CONCLUSION AND RECOMMENDATIONS

Symptom management self-efficacy and functional status of women undergoing breast surgery were adversely affected in our study. In the study, symptom management self-efficacy was found to be high in young, highly educated, married women who underwent conservative breast surgery. On the contrary, the functional life status was found to be low in those who were elderly and who had low educational status, advanced disease, and undergone mastectomy. A positive and significant correlation was found between symptom management self-efficacy and functional status. Health professionals should contribute to increasing the knowledge and skills of patients on symptom management by providing education, counseling, and life coaching to patients with problem-solving interventions. Arrangements that increase self-efficacy and functional capacity with a multidisciplinary approach should be included in routine patient care.
